# Seroprevalence of Human Cystic Echinococcosis in Alborz Province, Central Iran in 2015

**Published:** 2018-04

**Authors:** Hadi DABAGHZADEH, Amir BAIRAMI, Eshrat Beigom KIA, Mojgan ARYAEIPOUR, Mohammad Bagher ROKNI

**Affiliations:** 1. Dept. of Medical Parasitology and Mycology, School of Public Health, Tehran University of Medical Sciences, Tehran, Iran; 2. Dept. of Medical Parasitology and Mycology, School of Medicine, Alborz University of Medical Sciences, Karaj, Iran; 3. Center for Research of Endemic Parasites of Iran (CREPI), Tehran University of Medical Sciences, Tehran, Iran

**Keywords:** Hydatidosis, Epidemiology, ELISA, Iran

## Abstract

**Background:**

The aim of this study was to conduct a sero-epidemiological survey in Alborz Province, central Iran to detect the rate of hydatidosis in the city and nearby villages.

**Methods:**

Overall, 680 serum samples were collected from 536 male and 127 female subjects referred to different health centers of Karaj, Alborz Province, central Iran and nearby villages in 2014–15. All patients filled out a questionnaire and an informed consent. Sera were analyzed using indirect-ELISA test with AgB. Ten μg/ml antigens (Proceeded hydatid fluid), serum dilutions of 1:500 and conjugate anti-human coombs with 1:10000 dilutions were utilized to perform the test. Data analysis was conducted using SPSS software ver. 11.5.

**Results:**

Twenty-three cases (3.4%) were positive for hydatidosis by ELISA test. The prevalence of hydatidosis among females and males was 3.1% and 4.7%, respectively. The rate of the disease was significantly higher in areas where dogs were higher (*P*<0.05). There was no significant difference as regards age groups, sex, job, residency, and literacy. Regarding occupation, housekeepers had the highest rate of infection as 5.9%. The seroprevalence of infection was 4.2% in bachelors and master people which showed the highest rate. As regards residency, urban life showed no significant difference with rural life (2.8% vs. 4.4%). Age group of 30–39 yr old, with 4.3% as prevalence had the highest rate of positivity (*P*>0.05).

**Conclusion:**

Because of the specific situation of Alborz Province, and availability of many stray dogs, obtained rate of hydatidosis shows that the authorities should be cautious to monitor the disease.

## Introduction

Cystic echinococcosis (CE) or hydatidosis, caused by the larval stage of *Echinococcus granulosus*, is regarded as one of the most important zoonotic helminthic diseases globally. Not only human but also a broad spectrum of animal are infected with the disease as well. In human it occurs after ingestion of parasite’s egg through different ways including eating contaminated vegetable, contact with dogs, etc. (1, 2).

Human CE is endemic in some countries of the Mediterranean region, including Iran. In Iran, CE is one of the most important parasitic infections of humans and domestic animals (3–5). Alborz Province located in central Iran encompasses a unique situation due to issue arisen after war.

Many stray dogs are dispersed in the region of no control over them. The slaughtering of livestock without veterinary control, eating vegetables without washing with salt and detergent, and drinking water without filtration are among other problems of the region (6). These factors ease the matrix for spreading hydatidosis.

We aimed in this study to determine the seroprevalence of human hydatid infection by ELISA using AgB and to find out association of risk factors for acquisition of this infection in Alborz Province populations.

## Materials and Methods

### Study Area

**Fig. 1:**
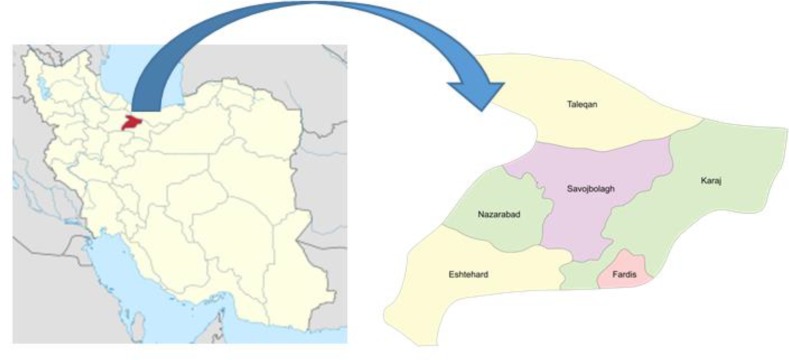
The geographic location of Alborz Province, 35 km west of Tehran

Alborz Province is situated between 35 degree and 48 degree north latitude, and 50 degree plus 58 degree east longitude (Fig. 1). It has 7 counties as follows: Karaj, Savojbolagh, Taleghan, Nazarabad, Fardis, Eshtehard and Mahdasht. Alborz Province is situated 35 km west of Tehran, at the foothills of the Alborz Mountains, and is the Iran’s smallest province in area. According to the national census, population of Alborz was 2,412,513 in 2011 out of which 90.5% lived in urban areas (http://againstallodds.wikia.com/wiki/Alborz_Province).

### Samples

The study population was 680 asymptomatic individuals selected randomly from 6 villages of 7 counties including Najmabad, Mahdasht, Taleghan, Sira, Chaharbagh, and Hyve as well as Karaj county in Alborz Province, central Iran using systematic random sampling in 2014–2015. For calculating the needed sample, we used expected prevalence of 0.05% at a confidence level of 95%.

Informed consent was taken from the participants before sampling and Ethics Committee of Tehran University of Medical Sciences approved the study.

A detailed demographic characteristics and relevant history was recorded (e.g. age, gender, place of residence, contact with dogs etc.). In addition, sera from 30 healthy blood donors, selected randomly from donors in Karaj Blood Transfusion Organization (KBTO) were included to find out baseline titer and to calculate cut-off OD (optical density).

After taking serum samples, they were sent to the Department of Medical Parasitology and Mycology, School of Public Health, Tehran University of Medical Sciences under cold situation for further examination.

### Antigen

Hydatid cyst fluid (HCF Ag) was collected from hydatid cysts of the livers and lungs of sheep slaughtered at the local abattoirs of Tehran. Antigen B was prepared and purified from HCF as described earlier (7).

### ELISA test

Microplate wells were coated over night at 4 °C with 100 μl AgB (20 μg/ml) in 0.05 M bicarbonate buffer, pH 9.6 (8). Wells were washed 3 times in PBS plus 0.05% Tween 20 (PBS-T) and blocked with PBS-T containing 1% BSA for 30 min at 37 °C. Sera were added at 1:500 dilutions in PBST, incubated at 37 °C for 1 h then washed as before. Antihuman IgG-HRP (Sigma Chemical Co., Poole, Dorset, United Kingdom) conjugates were added at 1: 10000 dilutions in PBS-T and the microplate incubated and washed as before. This was then developed in OPD substrate (5 mg 1, 2 phenylenediamine, 12.5 ml of 0.2 M citrate phosphate buffer pH 5, 10 μl 30% H_2_O_2_). The absorbance was read at 492 nm after 10 min using an automatic microplate reader (State Fax® 2100, Awareness, USA).

### Statistical analysis

Cut-off was calculated as mean + 3 SD. Data were analyzed using SPSS Ver. 11.5 (Chicago, IL, USA). Significant differences were accepted when the *P* value was <0.05.

## Results

**Fig. 2:**
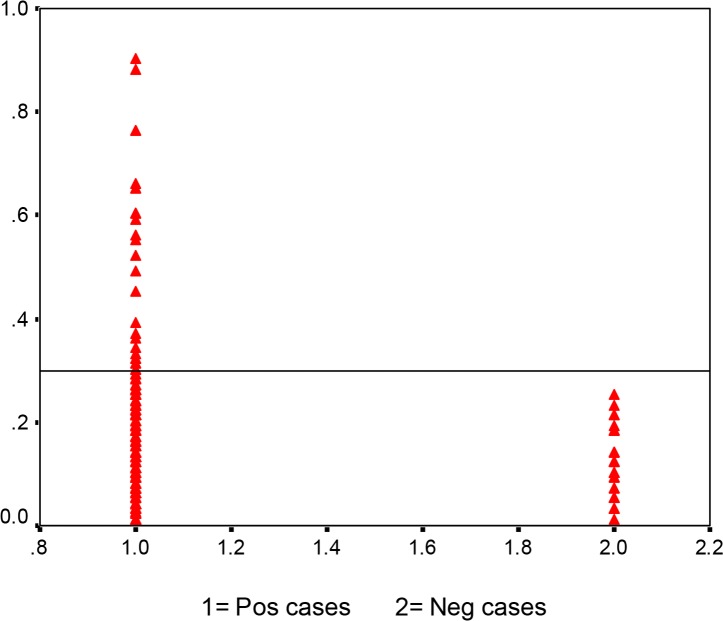
Analysis of sera from subjects and normal controls by IgG-ELISA. Serum samples obtained from patients with hydatidosis (Lanes 1; n=680), normal controls (Lanes 2; n=30)

Out of 680 serum samples 23 (3.4%) were found positive for *E. granulosus* IgG antibodies. The descriptive characteristics of the samples in terms of gender, place of residence and age group, job, care of dog and study are presented in Table 1. Cut off was calculated as 0.29 (Fig. 2).

**Table 1:** Characteristics of people investigated by ELISA for human cystic echinococcosis in terms of age group

***Age group (yr)***	***Total No.***	***Seropositivity No. (%)***
10–19	11	0 (0.0)
20–29	185	6 (3.2)
30–39	234	10 (4.3)
40–49	149	4 (2.7)
50–59	87	3 (3.4)
60–90	14	0 (0.0)

Prevalence of antibodies and associated risk factors antibodies to *E. granulosus* were detected in 680 subjects aged 10–90 years. The seroprevalence rate was insignificantly higher among the people aged 30–39 yr (n = 234, 4.3%) than in older subjects (50–59 yr, n = 87, 3.4%; and ≥60 yr N = 14, 0%) (*P* = 0.89) (Table 1).

Seropositivity was more prevalent in females (70%) than in males (30%), but was not statistically significance (*P* = 0.41).

Several demographic characteristics (sector of residence, education of volunteers, and occupation) were so important associated with the presence of *Echinococcus* antibodies in analysis. There was no significant different between factors including occupation (*P* = 0.31), inhabitant (*P* = 0.26), gender (*P* = 0.41), ages (*P* = 0.89).

## Discussion

In this study, we detected the seroprevalence of human hydatidosis in Karaj, Alborz Province, central Iran using ELISA as 3.4%.

Hydatidosis has a broad spectrum dissemination globally with the highest prevalence in parts of the Russian Federation and adjacent independent states, China, north and Africa, Australia, South America, Kenya and turkey. It is most intensively endemic in parts of Spain, southern Italy, Sardinia, and in India (9). The prevalence rate of human CE has been recorded as 5%–10% in the endemic parts of Peru, Argentina, east Africa, central Asia, and China (10). In turkey as 18–20 cases per 100000 population (11), in southern Libya 6.8% (12), and 1–200 per 100000 in India in 2015 (13). In Iran, the prevalence of CE in Khorramabad, Lorstan Provonce during July–November 2011 was 15.4% (14), 13.8% in southwest of Iran (15), and 13.7% in southern Iran (16). The rate of infection in our study shows more or less a similar rate with other parts of Iran (3).

In this study, the highest rate of infection was in the age group of 30–39 yr (4.3%) which is similar to the result of Gharahdaghi et al. study (17). Previous studies have reported the 10–19 yr old as the highest infected age group in Zanjan (18), 60–80 yr old in Hamadan (19), and 20–40 yr old in Kurdistan (20). In addition, age groups of 20–30, 60–90 and 30–60 yr old have been reported as the highest rate of infection in Kerman, Meshkinshahr and Qom, respectively (21–23). Each region of Iran has its specific situation in terms of ecology, economy and culture, so it is would be obvious that we expect differences in this regard. The area in our study has a very broad compendium of races and population especially after Iraq-Iran war, so we notice a diverse population in Karaj.

In this study infected females were more prevalent than males but is was not significant (4.7% vs. 3.1%). In some studies in Iran males more than females (21, 22, 24). Instead, other studies showed that females were more infected than males, e.g., in Golestan Province (3.16% vs. 1.93%) (25), and in Zanjan, 3.2% vs. 2.7%

We noticed that people with higher education background like MSc holders showed the highest rate of infection (4.2%) which is in contrast with other studies claiming that lack of public health education were the main reasons for the high prevalence (26).

As for occupation most studies in Iran have reported that homemakers were more infected than others (4, 18, 25). This is because of more contact with parasite egg by homemakers like washing vegetables, cleaning soil yards in villages, etc.(3).

The limitation of this study might be mentioned as need for investigation more population due to broad scope of people living in Karaj area but shortage of budget and time inhibited us to resolve this problem, although this is the first report of CE in this region and we believe it could help policy makers to have better feature of the problem there.

## Conclusion

The rate of prevalence in Alborz Province shows that in this region, we have a potential danger of spreading the daises like many other regions of Iran, hence more health measurements should be taken into account. Considering the broad scope of races and population in this area, it is expected that health authorities consider seriously CE there.

## Ethical considerations

Ethical issues (Including plagiarism, informed consent, misconduct, data fabrication and/or falsification, double publication and/or submission, redundancy, etc.) have been completely observed by the authors.
